# Soil-specific enzyme activity provides novel insight into the soil microbial necromass accumulation during sand dune fixation

**DOI:** 10.3389/fmicb.2025.1687297

**Published:** 2025-09-26

**Authors:** Qing Qu, Xuying Hai

**Affiliations:** ^1^Sichuan Philosophy and Social Key Laboratory of Monitoring and Assessing for Rural Land Utilization, School of History, Geography and Tourism, Chengdu Normal University, Chengdu, China; ^2^Institute of Soil and Water Conservation, Chinese Academy of Sciences and Ministry of Water Resources, Yangling, China; ^3^School of Forestry and Prataculture, Ningxia University, Yinchuan, Ningxia, China

**Keywords:** degeneration, soil enzyme activity, grassland, recovery, microorganisms, soil nutrients

## Abstract

**Introduction:**

Soil enzymes are critical to plant growth and soil carbon turnover. However, the traditional method of assessing enzyme activity per unit of soil may be insufficient; instead, soil-specific enzyme activity per unit of soil organic carbon (SOCE) or microbial biomass carbon (MBCE) has been widely used to characterize soil carbon accumulation.

**Methods:**

We systematically examined the changes in SOCE and MBCE with sand dune fixation (mobile, semi-mobile, semi-fixed, and fixed). We explored the implications of this soil-specific enzyme activity for soil microbial necromass carbon (NC) and soil organic carbon (SOC) accumulation.

**Results:**

We found that β-1, 4-glucosidase, β-D-cellobiosidase, β-1, 4-N-acetylglucosaminidase, and L-leucine aminopeptidase in SOCE and MBCE, the soil enzyme activity coefficient (SEAC), and the geometric mean of enzyme activity (GMEA) were significantly higher in semi-mobile, semi-fixed, and fixed dunes than those in mobile dunes. Furthermore, SOCE, MBCE, SEAC, and GMEA showed significant relationships with microbial NC and SOC. Specifically, soil-specific enzyme activity accounted for 32.2 and 24.1% of microbial NC and SOC variance, respectively.

**Conclusion:**

Dune fixation significantly increases SOCE and MBCE. More importantly, we recommend that changes in SOCE and MBCE should be widely used to assess microbial NC and SOC accumulation in degraded sandy land ecosystems.

## 1 Introduction

Desertification is an important ecological and environmental problem that severely restricts human survival and threatens sustainable economic and social development (Kéfi et al., 2007; Peters et al., 2012). The Mu Us Sandy Land is an important area for ecological restoration (Li et al., 2017). Since the 1950s, the ecosystem in this region has been severely degraded because of overgrazing (Miao et al., 2016). Local restoration measures, such as fencing, cropland abandonment, and vegetation restoration, have been implemented to restore the ecological environment, which has significantly changed the land-use pattern and affected plant growth and material cycling processes in the local ecosystem (Guo et al., 2023). Currently, mobile (coverage: <5%), semi-mobile (5–20%), semi-fixed (21–50%), and fixed (>50%) dunes with different vegetation coverages have formed (Chen et al., 2022).

Currently, further research has focused on soil absolute enzyme activity (SAE; Nannipieri et al., 2012; Zheng et al., 2025), but in natural soil, enzymes coexist with soil microorganisms and carbon (Stone et al., 2014; Xu et al., 2020). Therefore, soil enzymes cannot be separated from organic matter or microorganism (Liu et al., 2017). Based on this relationship, researchers have introduced the concept of soil-specific enzyme activity [per unit of soil organic carbon (SOCE) or microbial biomass carbon (MBCE)] (Yu et al., 2019). SOCE and MBCE reflect the activity based on organic matter and microorganisms, which providing clear insights into changes in SOC and MBC (Raiesi and Beheshti, 2014).

Some scholars have noted that SOCE and MBCE are regulated by land-use change (Xiao et al., 2021). For example, Zhang et al. (2015) reported consistent changes in both SOCE and SAE in response to different fertilization treatments. Raiesi and Salek-Gilani (2018) found that after cropland abandonment, SAE increased, whereas SOCE decreased with recovery time. The roles of SOCE and MBCE also differ in their responses to environmental changes. For example, Raiesi and Beheshti (2014) indicated that MBCE and SOCE respond consistently to land-use changes. However, Xu et al. (2020) observed that SOCE first increased and then tended to stabilize with recovery time, whereas MBCE gradually decreased with longer recovery times. Consequently, changes in SOCE and MBCE with restoration remain inconclusive.

Soil enzymes are sensitive to sand dune fixation (ecological restoration) (Cao C. et al., 2024). It is generally accepted that sand dune fixation increases plant diversity and vegetation cover (Qiao et al., 2012), which in turn produces more litter that returns to the soil, thereby increasing soil nutrient content and providing more food resources for soil microorganisms. This resource increase boosts enzyme secretion by microorganisms (Cheng et al., 2022). Moreover, sand dune fixation promotes plant growth and microbial activity, thereby promoting interactions among plants, microorganisms, and soil development (Cao et al., 2017; Alamusa et al., 2023). In arid and semi-arid regions, soil water controls microbial activity (Li et al., 2023). By increasing vegetation cover, dune fixation reduces water evaporation and enhances soil water availability (Cao M. et al., 2024). Increases in soil stability and water content further enhance SAE (Xu et al., 2016), but the effect of sand dune fixation on SOCE and MBCE remain poorly understood.

Soil microorganisms regulate soil carbon transformation, in the form of living organisms and microbial necromass carbon (NC; Buckeridge et al., 2022; Xiang et al., 2024). Liang et al. (2019) confirmed that microbial NC, as a stable soil carbon component, contributes more than 50% of SOC accumulation. This process occurs as soil microorganisms continuously grow, metabolize, proliferate, and die, leaving behind cell wall residues (Buckeridge et al., 2022). Chitin from fungal cell walls and peptidoglycan from bacterial cell walls accumulate in soils, which directly contribute to the soil carbon pool (Camenzind et al., 2023). Moreover, Xu et al. (2020) showed that soil-specific enzyme activity may help explain changes in carbon stability. However, the relationship between SOCE, MBCE, and microbial NC, as well as whether SOCE and MBCE can explain changes in microbial NC, requires further exploration.

To explore the correlation between SOCE and MBCE with soil microbial NC during dune fixation, we established study sites with varying dune coverages: mobile, semi-mobile, semi-fixed, and fixed dunes. We aimed to study the effect of dune fixation on SOCE and MBCE and its relationship with plant and microbial community characteristics. We hypothesized that dune fixation would lead to increased SOCE and MBCE. Additionally, we hypothesized that the SOCE and MBCE are significantly positively correlated with microbial NC.

## 2 Methods

### 2.1 Study site

**Figure 1:**
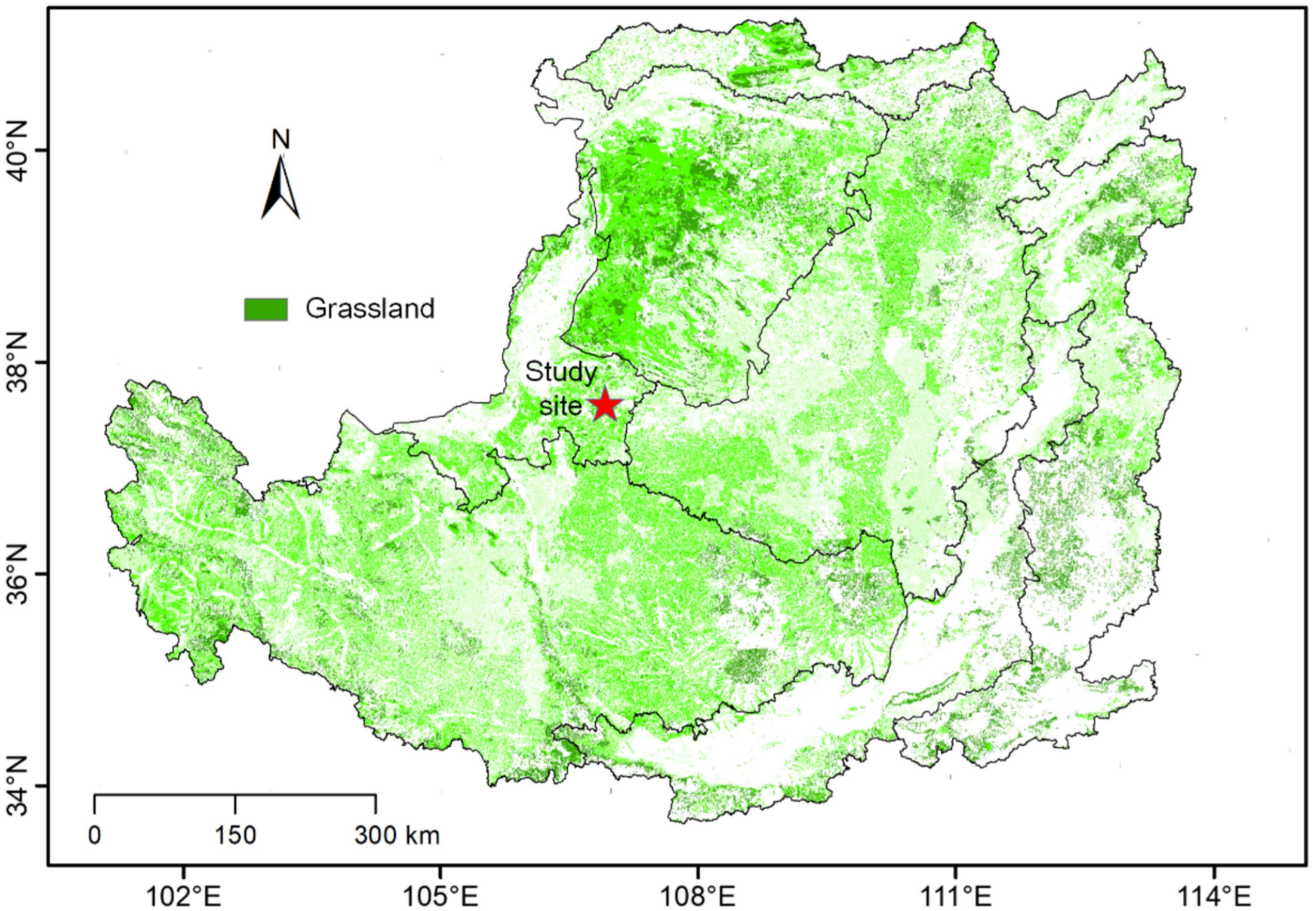
The location of the study site.

The study site is located in Yanchi County, Ningxia Hui Autonomous Region, China (Figure 1). The mean annual precipitation and temperature are 250–350 mm and 6.0 °C–8.5 °C, respectively, and the elevation is 1,200–1,600 m. The wind is mainly northwesterly, with annual average wind speeds of 2.1–3.3 m.s^−1^, especially from March to May. The soil type is typical eolian sand soil, with loose surface material, rich sand source material, and strong eolian sand activity. The zonal vegetation primarily consists of *Agriophyllum squarrosum*, *Artemisia ordosica*, *Aster altaicus*, *Artemisia scoparia*, and *Caragana microphylla*. In the 1950s, the ecosystem of the region was severely degraded owing to overgrazing. Consequently, fencing has been implemented to restore these degraded ecosystems, and grassland communities at different stages of restoration have been formed.

### 2.2 Experimental setting and sample analysis

Four treatments were established: mobile, semi-mobile, semi-fixed, and fixed dune groups. Three 5 × 5 m plots were set up for each dune type, totaling 12 plots. The slope angle, slope direction, and slope position in each treatment plot remained unchanged. In each plot, three 1 × 1 m quadrats were set evenly along the diagonal, totaling 36 quadrats. A vegetation diversity survey was conducted for each sample quadrat. The soil depth is 0–20 cm. The visible stones and roots in soil samples were removed and sieved using a 2-mm sieve and then divided into three parts: one part was used to determine the microbial community; the second, to determine the enzyme activity; and the third, to determine the microbial NC content.

Microbial community and microbial NC were determined by the Rhonin Biosciences^1^ and Baihui Organisms^2^ companies, respectively. Soil enzyme activity of β-1, 4-glucosidase (BG), β-D-cellobiosidase (CBH), β-1, 4-N-acetylglucosaminidase (NAG), L-leucine aminopeptidase (LAP), and acid phosphatase (AP) were determined using 96-microplate enzymic fluorescence assays (German et al., 2011). The soil microbial biomass was determined by chloroform fumigation methods (Vance et al., 1987). The soil pH, organic carbon (SOC), total nitrogen (TN), and total phosphorus (TP) were determined using the pH meter, Walkley and Black, Kjeldahl, and molybdenum blue methods, respectively (Bremner, 1982; Nelson and Sommers, 1982). The detailed methods of soil microbial community, microbial NC, soil microbial biomass, and enzyme activity were described in Zhou et al. (2025).

### 2.3 Statistical analyses

The Shannon–Weiner diversity, Pielou evenness, and Margalef richness indices were selected to characterize plant diversity, which were calculated as:

(1) $$\text{Shannon}–\text{Weiner diversity index}=-\sum P_{i}\log P_{i}\;(i=1,2,3,\cdots ,S)$$

(2) $$\begin{array}{l} \text{Pielou evenness index}=\text{Shannon}–\\ \text{Weiner diversity index}/\log(S) \end{array}$$

(3) Margalef richness index=S

where *Pi*, *S*, and *N* are the proportion of species i, number of species, and total number of individuals, respectively.

The SOCE and MBCE were calculated as follows (Trasar-Cepeda et al., 2008):

(4) $$\text{SOCE}/\text{MBCE}=\frac{\text{Soil absolute enzyme activity}}{\mathrm{SOC}/\mathrm{MBC}}$$

The soil enzyme activity coefficient (SEAC) reflects the relative microbial demand for carbon and nitrogen, which was calculated as:

(5) $$\text{SEAC of}\;\mathrm{BG}\;\text{and}\;\mathrm{CBH}=\frac{\mathrm{BG}/\mathrm{CBH}}{\mathrm{MBC}}$$

(6) $$\text{SEAC of}\;\mathrm{NAG}\;\text{and}\;\mathrm{LAP}=\frac{\mathrm{NAG}/\mathrm{LAP}}{\mathrm{MBN}}$$

The GMEA was calculated as follows (Hinojosa et al., 2004):

(7) $$\text{GMEA}=\sqrt[{5}]{\mathrm{BG}\times \mathrm{CBH}\times \mathrm{NAG}\times \mathrm{LAP}\times \mathrm{AP}}$$

Microbial NC was calculated as follows:

(8) BacterialNC=MurA×45

(9) $$\text{Fungal}\;\mathrm{NC}=(\frac{\text{GluN}}{179.17}-\frac{\text{MurA}}{251.23}\times 2)\times 179.17\times 9$$

(10) MicrobialNC=BacterialNC+FungalNC

where, MurA was muramic acid; GluN was glucosamine.

The results of plant diversity and soil physicochemical properties in different dunes are shown in Supplementary Tables S1, S2. SOCE, MBCE, soil nutrients, and plant and microbial diversity were analyzed using a one-way analysis of variance. The Pearson’s correlation was used to evaluate the relationships of SOCE and MBCE with microbial NC, SOC, soil nutrients, and plant and microbial diversity. Random forest analysis was used to assess the relative contributions of SOCE, MBCE, soil nutrients, and plant and microbial diversity to microbial NC and SOC (randomForest package in R version 4.5.0).

## 3 Results

### 3.1 Dynamics of SOCE and MBCE

**Figure 2:**
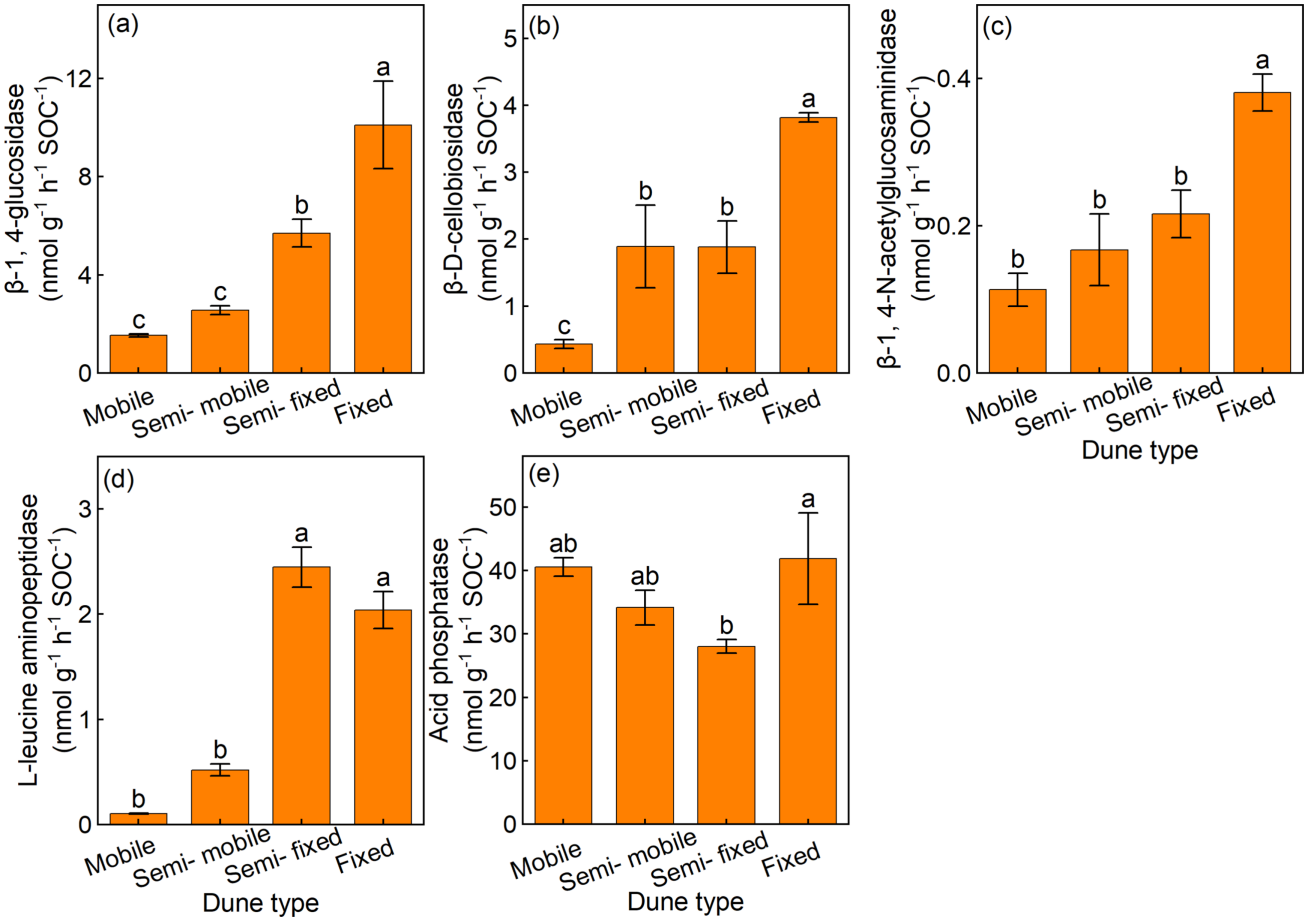
Mean (±) of soil-specific enzyme activity per unit of soil organic carbon in **(a)** β-1, 4-glucosidase (BG/SOC), **(b)** β-D-cellobiosidase (CBH/SOC), **(c)** β-1, 4-N-acetylglucosaminidase (NAG/SOC), **(d)** L-leucine aminopeptidase (LAP/SOC), and **(e)** acid phosphatase (AP/SOC) of different dune types.

The semi-fixed and fixed dunes had higher BG/SOC and LAP/SOC, compared with the semi-mobile and mobile dunes (Figures 2a,d). Furthermore, the fixed dunes had higher CBH/SOC, and NAG/SOC than the semi-mobile and mobile dunes, and the fixed dunes also had higher AP/SOC than other dune types (Figures 2b,c,e).

**Figure 3:**
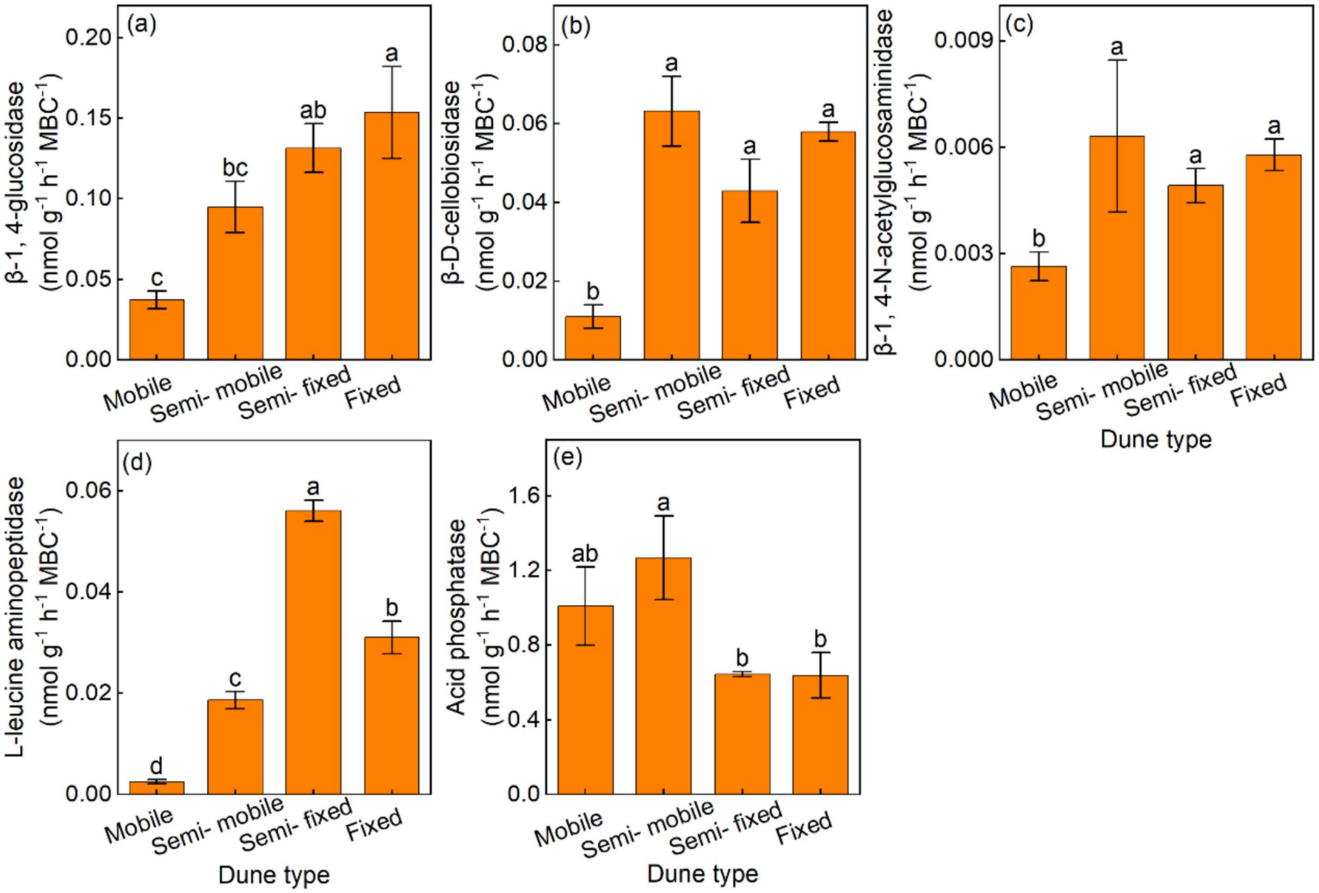
Mean (±) of soil-specific enzyme activity per unit of microbial biomass carbon in **(a)** β-1, 4-glucosidase (BG/MBC), **(b)** β-D-cellobiosidase (CBH/MBC), **(c)** β-1, 4-N-acetylglucosaminidase (NAG/MBC), **(d)** L-leucine aminopeptidase (LAP/MBC), and **(e)** acid phosphatase (AP/MBC) of different dune types.

Additionally, the semi-fixed and fixed dunes had higher BG/MBC and LAP/MBC, compared with the semi-mobile and mobile dunes (Figures 3a,d). The CBH/MBC and NAG/MBC were lowest in mobile dunes, and the semi-fixed and fixed dunes had lower AP/MBC, compared with the semi-mobile and mobile dunes (Figures 3b,c,e).

### 3.2 Dynamics of the SEAC and GMEA

**Figure 4:**
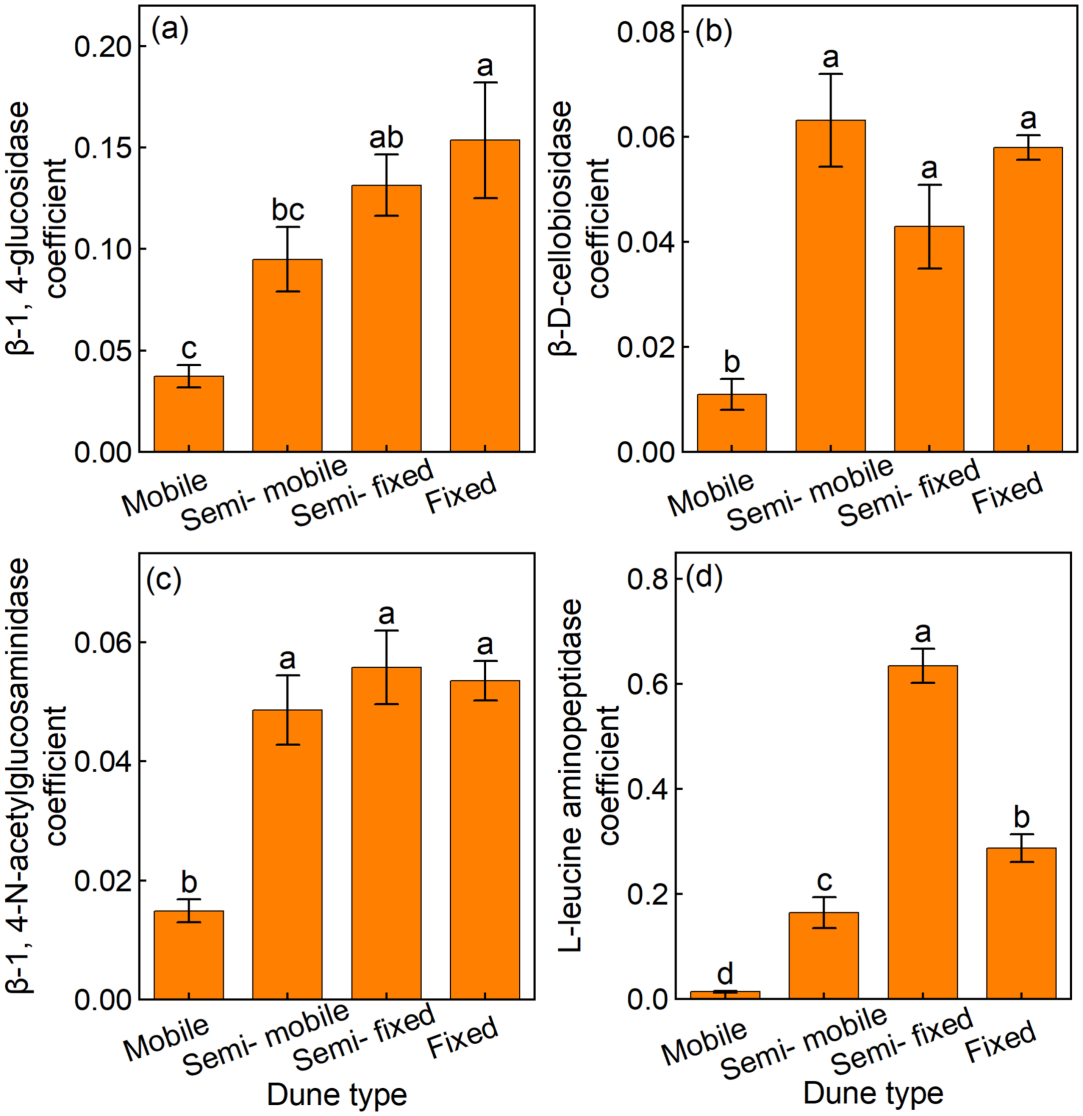
Mean (±) of soil enzyme activity coefficient in **(a)** β-1, 4-glucosidase (BG), **(b)** β-D-cellobiosidase (CBH), **(c)** β-1, 4-N-acetylglucosaminidase (NAG), and **(d)** L-leucine aminopeptidase (LAP) of different dune types.

**Figure 5:**
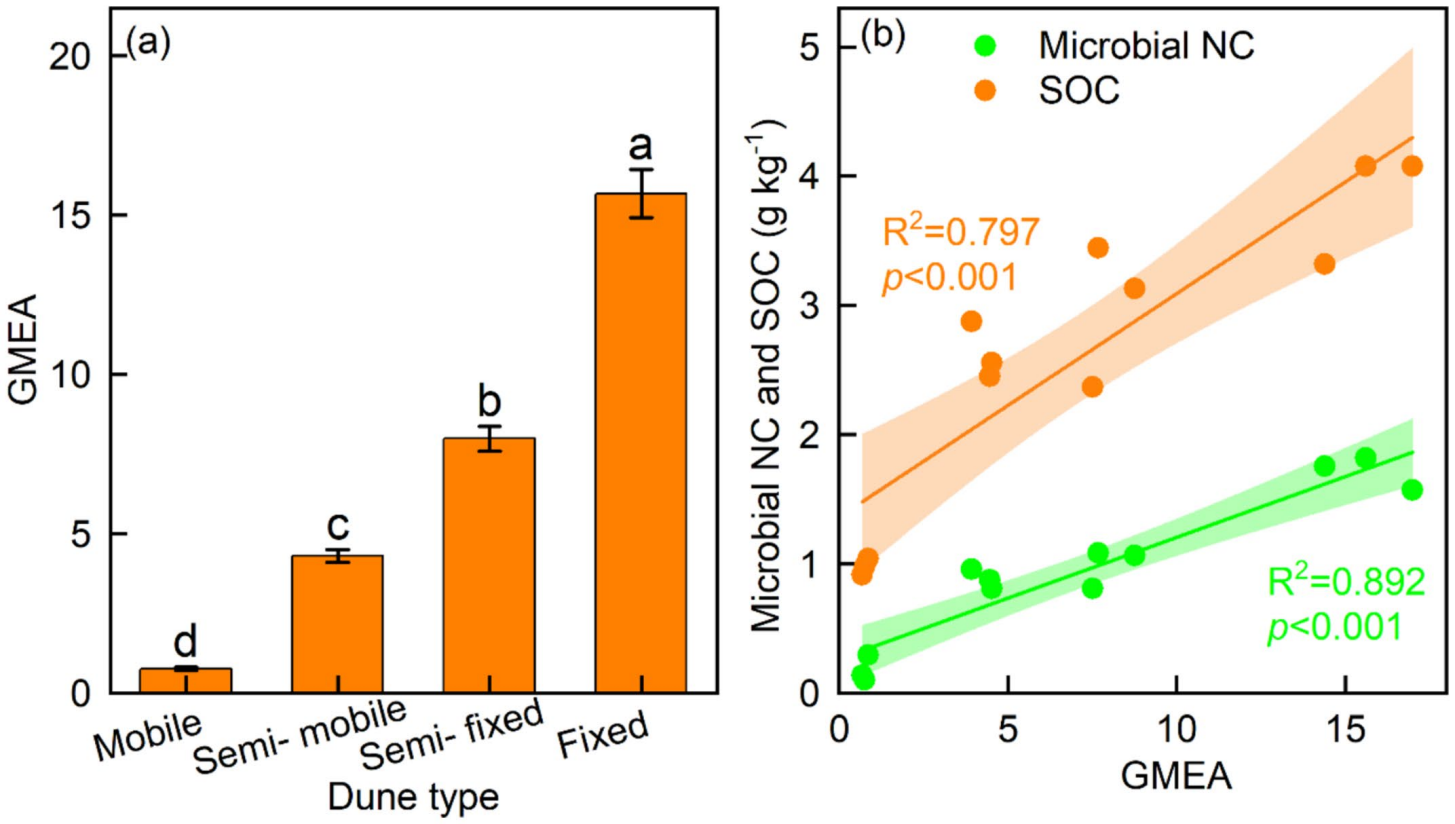
Mean (± SE) of **(a)** geometric mean of enzyme activity (GMEA) and **(b)** relationship of GMEA with soil microbial necromass carbon (NC) and soil organic carbon (SOC).

The fixed dunes had higher BG coefficients than other dune types, whereas the mobile dunes had lower CBH, NAG, and LAP coefficients than other dune types (Figures 4a–d). The GMEA of the fixed dunes was approximately twice that of the semi-fixed dunes, four-fold that of the semi-mobile, and 20 times that of the mobile dunes (Figure 5a).

### 3.3 Correlations of soil-specific enzyme activity with plant and soil variables

The SOCE was positively correlated with the MBCE and the enzyme activity coefficients overall. The SOCE and MBCE were positively correlated with the plant diversity indices (i.e., the Shannon–Weiner diversity, Pielou evenness, and Margalef richness indices), soil nutrients (i.e., TN and TP), and soil microbial properties (i.e., MBC, MBN, bacterial diversity, bacterial richness, fungal diversity, and fungal richness) (Table 1).

**Table 1:** Pearson’s correlation of soil extracellular enzyme activity coefficient with plant and soil variables.

Variables	BG/SOC	CBH/SOC	NAG/SOC	LAP/SOC	BG/MBC	CBH/MBC	LAP/MBC	BG coe	CBH coe	NAG coe	LAP coe	GMEA
CBH/SOC	0.82**											
NAG/SOC	0.90**	0.78**										
LAP/SOC	0.71**	0.64*	0.64*									
BG/MBC	0.88**	0.68*	0.76**	0.72**								
CBH/MBC		0.78**			0.60*							
NAG/MBC			0.60*		0.59*							
LAP/MBC				0.93**	0.67*							
AP/MBC	−0.60*			−0.63*								
BG coe	0.88**	0.68*	0.76**	0.72**	0.99**	0.60*	0.67*					
CBH coe		0.78**			0.60*	0.99**		0.60*				
NAG coe	0.82*	0.59*	0.67*	0.72**	0.76**	0.66*	0.74**	0.76**	0.66*			
LAP coe				0.89**			0.97**			0.69*		
GMEA	0.95**	0.89**	0.91**	0.75**	0.83**			0.83**		0.67*		
Plant D	0.86**	0.82**	0.79**	0.77**	0.88**	0.68*	0.66*	0.88**	0.68*	0.83**	0.60*	0.93**
Plant R	0.69*	0.79**	0.70*	0.75**	0.82**	0.83**	0.72**	0.82**	0.83**	0.93**	0.64*	0.80**
Plant E	0.80**	0.85**	0.79**	0.82**	0.84**	0.75**	0.72**	0.84**	0.75**	0.88**	0.65*	0.91**
Soil M	0.88**	0.84**	0.89**	0.79**	0.85**	0.58*	0.62*	0.85**	0.58*	0.75**		0.96**
pH	−0.59*	−0.67*			−0.60*	−0.62*		−0.60*	−0.62*	−0.63*		−0.68*
TN	0.75**	0.81**	0.71**	0.82**	0.84**	0.79**	0.77**	0.84**	0.79**	0.91**	0.71**	0.82**
TP				0.66*	0.69*		0.76**	0.69*		0.79**	0.75**	
MBC	0.91**	0.86**	0.87**	0.69*	0.71**			0.71**		0.58*		0.98**
MBN	0.93**	0.81**	0.93**		0.73**			0.73**				0.94**
Bacteria R	0.92**	0.85**	0.89**	0.61*	0.71**			0.71**				0.94**
Bacteria D	0.92**	0.84**	0.87**	0.80**	0.81**		0.59*	0.81**		0.62*		0.94**
Fungi R	0.84**	0.77**	0.77**	0.81**	0.80**		0.65*	0.80**				0.90**
Fungi D	0.67*			0.84**	0.66*		0.0.74**	0.66*			0.71**	0.77**

**p < 0.01, *p < 0.05.

### 3.4 Correlations of soil-specific enzyme activity with microbial NC and SOC

**Figure 6:**
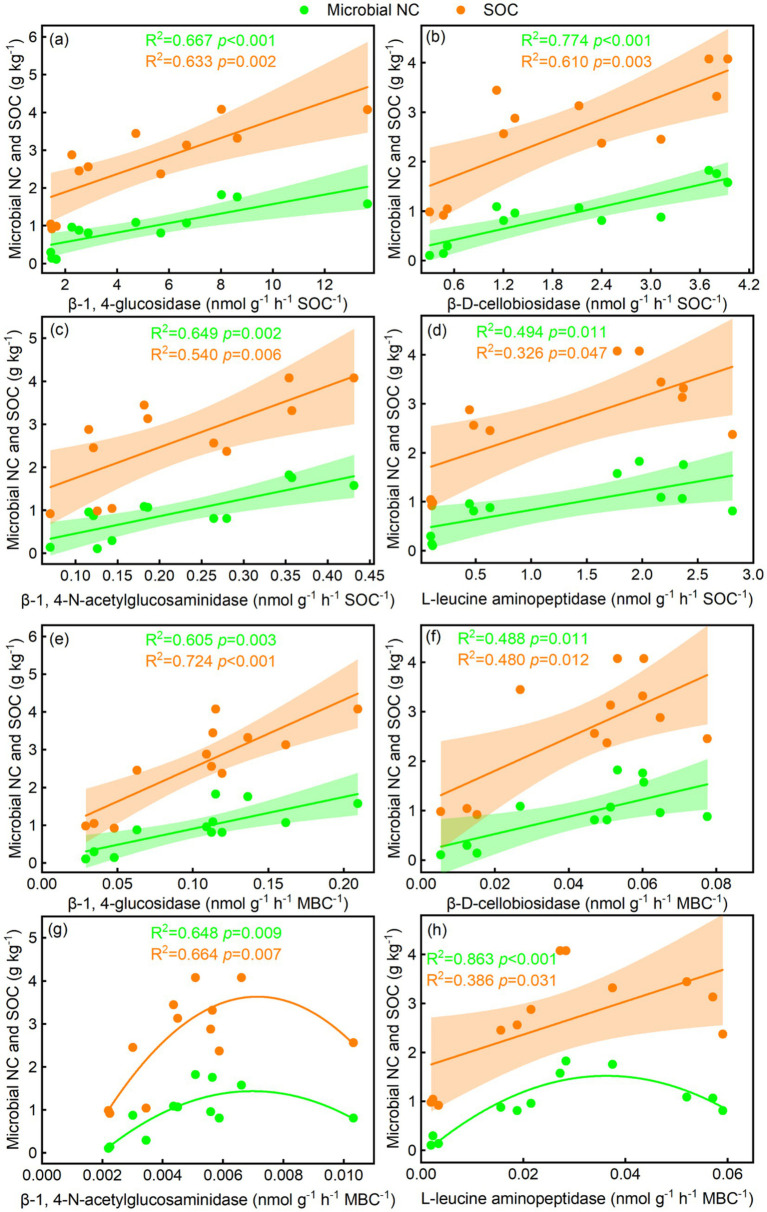
Relationships of soil-specific enzyme activity per unit of **(a–d)** soil organic carbon and **(e–h)** microbial biomass carbon with soil microbial necromass carbon (NC) and SOC.

**Figure 7:**
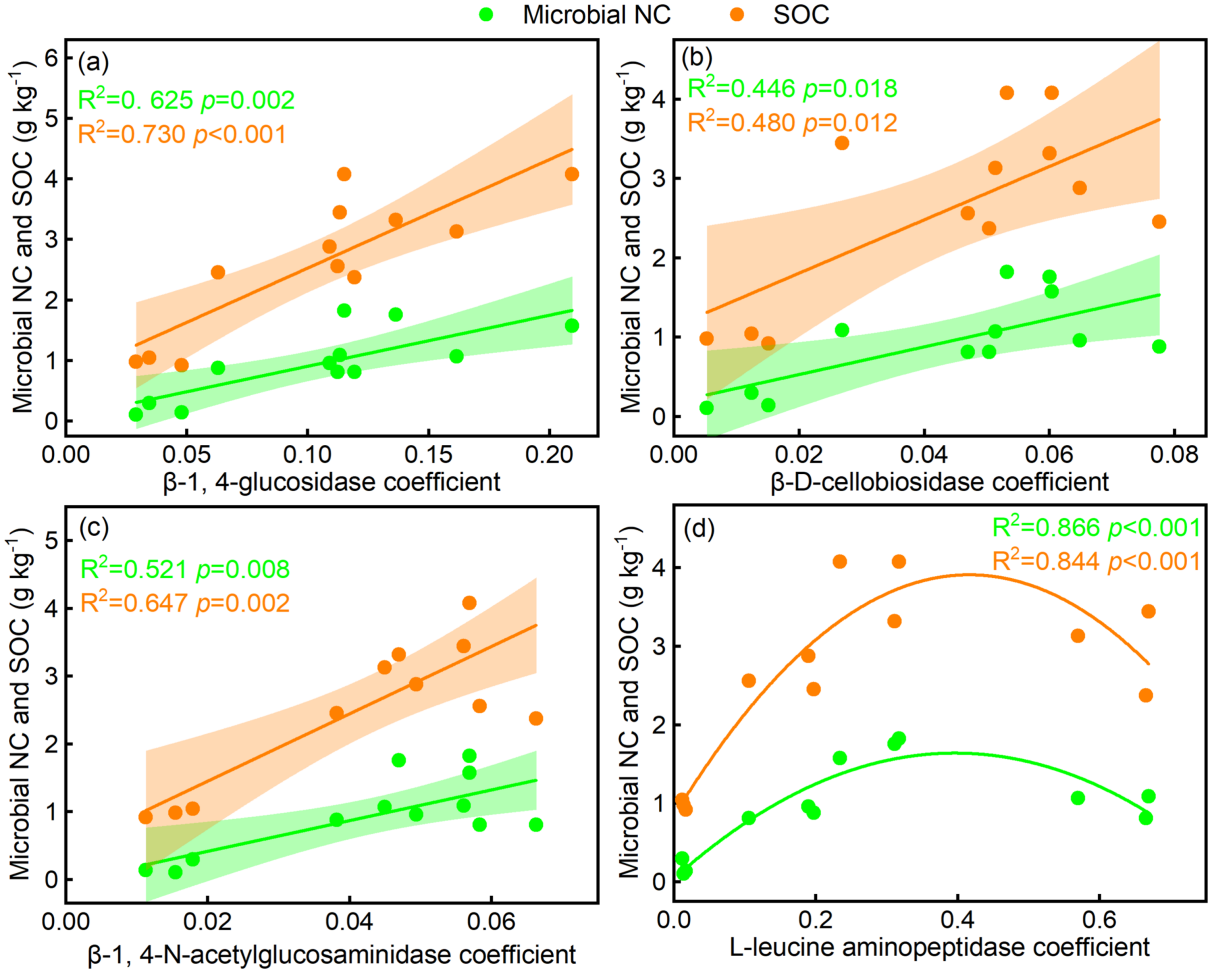
Relationships of soil enzyme activity coefficient with **(a–d)** soil microbial necromass carbon (NC) and soil organic carbon (SOC).

The BG/SOC, CBH/SOC, NAG/SOC, LAP/SOC, BG/MBC, and CBH/MBC were linearly related to microbial NC and SOC, whereas NAG/MBC was quadratically related to microbial NC and SOC (Figures 6a–g). LAP/MBC was linearly related to SOC, whereas LAP/MBC was quadratically related to microbial NC (Figure 6h). The BG, CBH, and NAG coefficients were linearly related to microbial NC and SOC, while the LAP coefficient was quadratically related to microbial NC and SOC (Figures 7a–d). In addition, GMEA was linearly related to microbial NC and SOC (Figure 5b).

**Figure 8:**
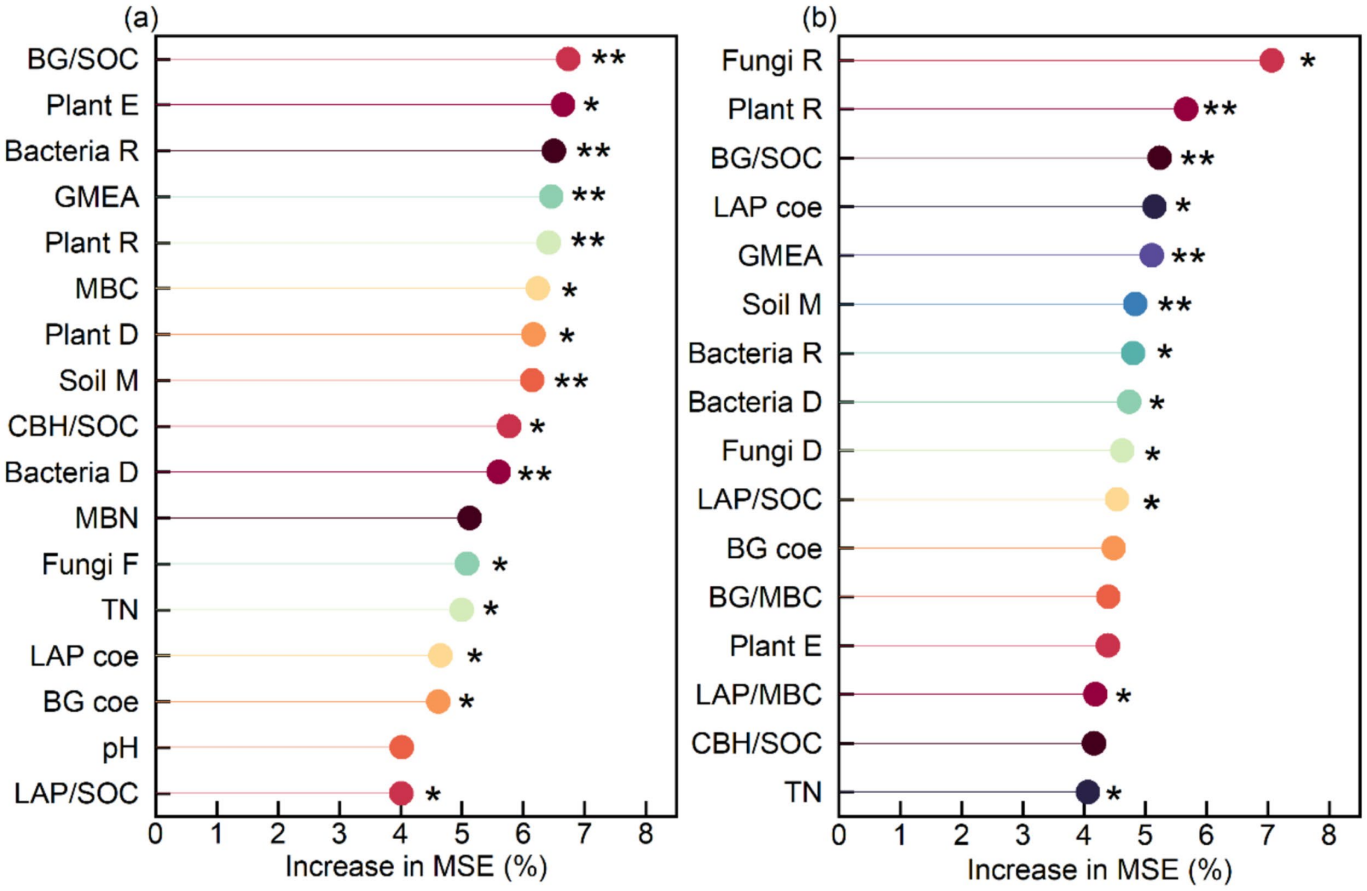
Main predictor importance (% of increase of MSE) of soil-specific enzyme activity and soil variables on **(a)** soil microbial necromass carbon and **(b)** soil organic carbon by random forest modeling analysis. **p < 0.01, *p < 0.05.

The microbial NC was mainly influenced by BG/SOC (6.7%), plant evenness (6.7%), bacterial richness (6.5%), GMEA (6.5%), plant richness (6.4%), MBC (6.2%), plant diversity (6.2%), soil moisture (6.1%), CBH/SOC (5.8%), bacterial diversity (5.6%), fungal richness (5.1%), TN (5.0%), the LAP coefficient (4.6%), the BG coefficient (4.6%), and LAP/SOC (4.0%), which cumulatively explained 86.0% of the variance in microbial NC (Figure 8a). The SOC was mainly influenced by fungal richness (7.1%), plant richness (5.7%), BG/SOC (5.2%), the LAP coefficient (5.1%), GMEA (5.1%), soil moisture (M) (4.8%), bacterial richness (4.8%), bacterial diversity (4.7%), fungal diversity (4.6%), LAP/SOC (4.5%), LAP/MBC (4.2%), and TN (4.1%), which cumulatively explained 59.9% of the variance in SOC (Figure 8b).

## 4 Discussion

### 4.1 Effect of dune fixation on SOCE

The traditional absolute enzyme activity (SAE) appears to be insufficient to characterize the accumulation of SOC (Xu et al., 2020). Consequently, SOCE has been widely used to characterize soil carbon turnover (Zhang et al., 2015; Xiao et al., 2021). As indicated by our hypothesis, sand fixation (ecological restoration) promoted an overall increase in SOCE (except for AP), indicating an increase in the enzyme production capacity of soil microorganisms. Although both SOC and soil absolute enzymes had positive effects on dune fixation, the percentage change in soil absolute enzymes (except AP) was higher than that of SOC, which led to an increase in SOCE.

There are three possible mechanisms for this: (1) Dune fixation increases plant diversity (Supplementary Table S2) and plant biomass (Qiao et al., 2012), which increases the production of dead leaves that enter the soil and provide energy and food for microorganisms (Cheng et al., 2022), thereby increasing SAE in the soil (Supplementary Table S2). (2) Differences in vegetation types caused by dune fixation significantly affect the connections between plant rhizospheres, thereby improving the soil porosity, soil bulk density, and aggregates (Alamusa et al., 2023). Furthermore, the increase in plant diversity and coverage leads to the secretion of large amounts of organic matter from the root system, which improves the soil physical structure (Jones et al., 2004; Liu et al., 2005; Liu et al., 2009; Cao et al., 2017) and increases the microbial activity (Supplementary Table S2), enzyme secretion, and SOCE (Xu et al., 2020). (3) The plant cover acts as a shade for the soil surface and effectively reduces the impact of rain and wind erosion, thereby protecting the environmental conditions of the soil (Cao M. et al., 2024; Xu et al., 2024), and providing a stable environment for the secretion of soil enzymes, leading to a higher SOCE. In addition, we were surprised to find that the SOCE of phosphatase enzymes was different from that of carbon and nitrogen enzymes, showing that AP/SOC in semi-fixed dunes was lower than that in mobile and semi-mobile dunes. This may be attributed to the efficiency of the phosphatases released by microorganisms decreasing during the late recovery period (Li et al., 2018), whereas SOC continued to increase (Xu et al., 2021), thereby reducing the AP/SOC. Overall, this study provides direct evidence that SOCE is sensitive to dune fixation and shows potential as a sensitive index for characterizing SOC accumulation.

### 4.2 Effect of dune fixation on MBCE, SEAC, and GMEA

The percentage increase in MBC during dune fixation was smaller than that of enzyme activity (Supplementary Table S1), which, in turn, led to an increase in MBCE (except for AP), confirming our hypothesis. The MBCE characterizes the metabolic activity and catalytic efficiency of enzymes produced by microbial communities (Waldrop et al., 2000). The changes in MBCE were consistent with the changes in soil nutrient content (Supplementary Table S2), indicating that, with the accumulation of soil nutrients, the metabolic activity and enzyme production ability of soil microorganisms gradually increased. During dune fixation, changes in plant diversity and biomass, accompanied by changes in soil physical and chemical properties, are important causes of changes in soil enzyme activity (Cheng et al., 2022; Naeimi et al., 2023). The increase in plant productivity produces more litter, which is conducive to the improvement of soil nutrient accumulation (Taylor et al., 2024; Yang et al., 2024). Soils with adequate nutrients tend to have higher soil microbial diversity and activity, which in turn enhances enzyme secretion (Moharana et al., 2024; Zhang et al., 2024), thereby increasing the MBCE (Raiesi and Beheshti, 2015). This conclusion was confirmed by the positive correlations between MBCE and plant diversity, soil nutrient content, and microbial diversity (Table 1). However, other studies have indicated that ecological restoration reduces MBCE (Raiesi and Salek-Gilani, 2018; Xu et al., 2020). The reason for these differences may be related to differences in the ecological community types. The research of Raiesi and Salek-Gilani (2018) and Xu et al. (2020) investigated an area with relatively abundant rainfall and found that the growth and reproduction of plants and microorganisms were less affected by water (Ghorbani et al., 2023). By contrast, this study was conducted in an extremely arid environment (with an average annual rainfall of 250–350 mm), and the reproduction of microorganisms was limited by water, resulting in a more significant impact of ecological restoration on microorganisms (Chang et al., 2024; Zhao et al., 2024). This study indicated that dune fixation significantly increased the metabolic activity of soil microorganisms and increased the production of phosphatase enzymes; MBCE was a sensitive index reflecting the interaction between soil enzyme activity and microorganisms.

The soil enzyme activity coefficient and GMEA are common indices for integrating soil enzyme data and information and are also important indicators of soil quality (Hinojosa et al., 2004). Dune fixation significantly increased the soil enzyme activity coefficient and GMEA. The increase in the GMEA was mainly due to an increase in enzyme activity associated with increased dune fixation. As explained earlier, dune fixation increases plant diversity and productivity (Qiao et al., 2012), leading to increased litter production and root turnover, which, in turn, leads to increased soil organic carbon and nutrient content (Cao M. et al., 2024). The increase in the organic substrates available to microorganisms enhances the activity of soil microorganisms and accelerates the secretion of enzymes (Cao et al., 2017; Naeimi et al., 2023). Therefore, overall, dune fixation accelerated the production of soil enzymes in this arid ecosystem.

### 4.3 Relationships of soil-specific enzyme activity with microbial NC and SOC

Absolute soil enzymes can explain the accumulation of microbial NC and SOC (Bai et al., 2024; Raza et al., 2024). However, the precise relationships of soil-specific enzyme activity with microbial NC and SOC are still not fully understood. Our study found a significant correlation between SOCE, MBCE, the enzyme activity coefficient, and GMEA with microbial NC and SOC. Additionally, dune fixation promoted the accumulation of microbial NC and SOC (Supplementary Table S2), indicating that higher soil-specific enzyme activity supports this process.

Although earlier studies have identified microbial traits as major drivers of microbial NC accumulation (Han et al., 2024), our study indicated that soil-specific enzyme activity accounted for up to 32.2% of the variations in microbial NC, compared to 6.7% for BG/SOC. The contributions of plant diversity and microbial communities were 19.3 and 23.4%, respectively. For SOC, soil-specific enzyme activity, plant diversity, and microbial community composition accounted for 24.1, 5.7, and 21.2%, respectively. These observations suggest that soil-specific enzymatic activity is crucial for accumulating both microbial NC and SOC. We highlight two key mechanisms behind these results. First, an increase in soil-specific enzyme activity can modulate the hydrothermal conditions and physical structure of the soil (Bharti et al., 2024), which affects the accumulation process of soil carbon. Second, vegetation input directly influences the dynamic changes in SOC (Zhou et al., 2019). However, both the quantity and quality of plant litter affect how microorganisms use substrates (Shigyo et al., 2024), thus influencing SOC distribution (Wiesmeier et al., 2019). Higher soil-specific enzyme activity supports plant growth and promotes the accumulation of both aboveground and subsurface biomass (Yu et al., 2019), leading to more plant litter and further promoting the accumulation of microbial NC and SOC (Xiao et al., 2021). In addition, soil-specific enzyme activity enhances the carbon use efficiency of soil microorganisms through both direct effects (enhanced microbial activity) and indirect effects (affecting root growth and its secretions) (Liu et al., 2017). Therefore, in arid regions, dune fixation produces higher aboveground biomass and litter input, collectively promoting soil microbial NC and SOC accumulation through increased root growth and more active microbial processes.

## 5 Conclusion

Our study confirmed that dune fixation increased the soil-specific enzyme activity (including SOCE and MBCE), the enzyme activity coefficient, and GMEA; among them, the GMEA of the fixed dunes was approximately twice that of the semi-fixed dunes, four-fold that of the semi-mobile, and 20 times that of the mobile dunes. This was mainly attributed to the increase in plant diversity, plant biomass, soil moisture, and soil nutrients. Moreover, SOCE and MBCE were significantly correlated with microbial NC and SOC. Although soil microbial communities and plant diversity largely influenced microbial NC and SOC, soil-specific enzyme activity explained even more variation, accounting for 32.2% of microbial NC and 24.1% of SOC. Therefore, this study provides strong evidence that SOCE and MBCE are sensitive indicators in responses to dune restoration, making them useful for explaining changes in microbial NC and SOC during ecological restoration.

## Data Availability

The original contributions presented in the study are included in the article/Supplementary material, further inquiries can be directed to the corresponding author.

## Footnotes

^1^ http://rhonin-bio.com/

^2^ http://www.baihuitech.cn/
