# Supplementary material for: Soil-specific enzyme activity provides novel insight into the soil microbial necromass accumulation during sand dune fixation
Source: Front Microbiol. 2025 Sep 26;16:1687297. doi: 10.3389/fmicb.2025.1687297 (PMC12511058; doi:10.3389/fmicb.2025.1687297)
Supplement: Supplementary file 1 [file Supplementary_file_1.docx]

**Table S1** Percentage change in soil organic carbon (SOC), microbial biomass carbon (MBC), and enzyme activity.

|  | SOC (%) | MBC (%) | BG (%) | CBH (%) | NAG (%) | LAP (%) | AP (%) |
| --- | --- | --- | --- | --- | --- | --- | --- |
| Semi- mobile dune | 168.20 | 75.32 | 346.73 | 1059.02 | 296.36 | 1250.00 | 125.52 |
| Semi-fixed dune | 204.08 | 202.07 | 1025.57 | 1184.83 | 467.33 | 1250.00 | 108.97 |
| Fixed dune | 290.01 | 495.01 | 2500.20 | 3375.74 | 1229.49 | 1250.00 | 295.34 |

Notes: BG, β-1, 4-glucosidase. CBH, β-D-cellobiosidase. NAG, β-1, 4-N-acetylglucosaminidase. LAP, L-leucine aminopeptidase. AP, phosphatase.

**Table S2** Changes in plant diversity and soil physicochemical properties under different dune.

|  | Plant diversity | Plant evenness | Plant richness | Water content  (%) | pH | SOC  (g kg^-1^) | TN  (g kg^-1^) |
| --- | --- | --- | --- | --- | --- | --- | --- |
| Mobile dune | - | - | - | 3.50±0.23d | 8.99±0.08a | 0.98±0.04c | 0.04±0.00c |
| Semi- mobile dune | 0.74±0.08c | 0.50±0.00c | 0.76±0.01d | 5.07±0.47c | 8.83±0.03ab | 2.63±0.13b | 0.21±0.02b |
| Semi-fixed dune | 1.06±0.08b | 0.66±0.02b | 0.83±0.02c | 6.36±0.43b | 8.81±0.05b | 2.98±0.32b | 0.26±0.02a |
| Fixed dune | 1.49±0.15a | 0.88±0.01a | 0.98±0.01a | 8.28±0.28a | 8.75±0.02b | 3.82±0.25a | 0.29±0.01a |
|  | **TP**  **(g kg^-1^)** | **Microbial NC**  **(g kg^-1^)** | **BG**  **(nmol g^-1^ h^-1^)** | **CBH**  **(nmol g^-1^ h^-1^)** | **NAG**  **(nmol g^-1^ h^-1^)** | **LAP**  **(nmol g^-1^ h^-1^)** | **AP**  **(nmol g^-1^ h^-1^)** |
| Mobile dune | 0.20±0.02b | 0.18±0.06c | 1.50±0.08c | 0.42±0.07c | 0.11±0.03c | 0.10±0.00c | 39.63±0.49c |
| Semi- mobile dune | 0.29±0.01a | 0.88±0.04b | 6.70±0.35bc | 4.87±1.41b | 0.44±0.12bc | 1.35±0.10b | 89.37±5.81b |
| Semi-fixed dune | 0.34±0.03a | 0.99±0.09b | 16.88±2.17b | 5.40±0.82b | 0.62±0.02b | 7.17±0.26a | 82.81±5.95b |
| Fixed dune | 0.30±0.01a | 1.72±0.07a | 39.00±8.40a | 14.60±1.3a | 1.46±0.17a | 7.72±0.25a | 156.67±17.03a |
|  | **MBC**  **(g kg^-1^)** | **MBN**  **(g kg^-1^)** | **Bacteria richness** | **Bacteria diversity** | **Fungi richness** | **Fungi diversity** |  |
| Mobile dune | 42.56±7.96c | 7.34±0.83b | 857.34±17.88c | 6.06±0.02c | 258.62±19.49c | 3.45±0.15b |  |
| Semi- mobile dune | 74.61±12.16c | 8.72±1.48b | 898.04±1.97bc | 6.21±0.00c | 334.79±21.31c | 3.50±0.16b |  |
| Semi-fixed dune | 128.56±8.88b | 11.37±0.86b | 1010.96±17.97b | 6.51±0.02b | 482.15±38.72b | 4.39±0.13a |  |
| Fixed dune | 253.24±22.24a | 27.17±1.86a | 1537.14±67.43a | 6.90±0.13a | 626.64±61.70a | 4.46±0.21a |  |

Notes: SOC, soil organic carbon. TN, soil total nitrogen. TP, soil total phosphorus. BG, β-1, 4-glucosidase. CBH, β-D-cellobiosidase. NAG, β-1, 4-N-acetylglucosaminidase. LAP, L-leucine aminopeptidase. AP, phosphatase. MBC, soil microbial carbon. MBN, soil microbial nitrogen. Lowercase letters indicate significant differences at *p* < 0.05.
